# Central retinal artery occlusion or retinal stroke: a neurosonologist’s perspective

**DOI:** 10.3389/fneur.2024.1397751

**Published:** 2024-06-10

**Authors:** Saulius Taroza, Dalius Jatužis, Vaidas Matijošaitis, Saulius Raugelė, Jurgita Valaikienė

**Affiliations:** ^1^Laboratory of Behavioral Medicine, Neuroscience Institute, Lithuanian University of Health Sciences, Palanga, Lithuania; ^2^Klaipėda University Hospital, Klaipėda, Lithuania; ^3^Clinic of Neurology and Neurosurgery, Institute of Clinical Medicine, Faculty of Medicine, Vilnius University, Vilnius, Lithuania; ^4^Department of Neurology, Lithuanian University of Health Sciences, Kaunas, Lithuania; ^5^Faculty of Health Sciences, Klaipėda University, Klaipėda, Lithuania

**Keywords:** central retinal artery occlusion, retinal stroke, POCUS, spot sign, ophthalmology

## Abstract

In central retinal artery occlusion (CRAO) or retinal stroke, which is usually a vision-threatening condition, timely diagnosis is imperative to improve the chances of retinal preservation and to establish adequate secondary prevention measures. Even though retinal strokes have been traditionally assigned to the field of ophthalmology, while considering reperfusion therapy as the only way to avoid permanent vision loss, we suggest prompt evaluation of CRAO causes (primarily related to cardiovascular risk factors) performed by a well-organized interdisciplinary team (ophthalmologist and neurologist) in a neurovascular center with stroke expertise. Therefore, the most suitable adjunct method for rapidly diagnosing non-arteritic CRAO could be target transorbital ultrasound, performed by an experienced neurologist/neurosonologist in the stroke unit. Consequently, after an ophthalmological assessment, a final decision on thrombolytic therapy could be made. We accept that further research is obviously needed to determine whether transorbital ultrasound could replace ophthalmological investigation in the case of a suspected acute retinal stroke. We assert that retinal stroke requires interdisciplinary treatment in cooperation with neurologists and ophthalmologists, with an additive value for each to achieve the best results for the patient.

## 1 Introduction

Central retinal artery occlusion (CRAO) or retinal stroke usually develops within seconds, leaving an individual with a high probability of permanent retinal injury with loss of vision. Therefore, prompt investigation should be done for possible acute intervention (1). Most commonly, CRAO develops due to embolic material lodgement in the central retinal artery (non-arteritic CRAO) or rarely due to inflammation of the central retinal artery wall (arteritic CRAO). Both are associated with a high probability of life-threatening disorders.

According to epidemiological surveys, this condition is rare—incidence varies from 1 to 10 in 100,000 in the United States and Korea, respectively (1). Furthermore, as with other vascular diseases, the incidence rate of CRAO increases with age (2). It is worth mentioning that in countries with improved chronic disease control, such as Korea, retinal stroke incidence shows a decreasing trend over time (3).

This article discusses the importance of CRAO recognition in time and possible alternatives for the evaluation of this devastating disorder, including ophthalmological investigation and ultrasound application, together with a dispute over their drawbacks and advantages. In this article, the discussion will go step-by-step from a more detailed description of the importance of this acute condition, current treatment possibilities, and diagnostics to the neurosonologist’s perspective on CRAO treatment and etiological work-ups, along with the presentation of two clinical cases from the authors’ archives.

## 2 Why should we talk about retinal stroke?

CRAO causes acute monocular and painless vision loss, with a low probability (<20%) of vision restoration to a sufficient functional level in untreated cases (4). Owing to the permanence of vision loss, this condition is associated with the deterioration of depth perception and a constricted visual field, leading to impaired quality of life (5, 6). Currently, there are no randomized controlled trials that have been proven for CRAO treatment (7). Due to the limited retinal ischemia tolerance, there is hope for an evidence-based early administered treatment for visual restoration in the coming future. Controversy remains regarding how long the retina can survive after blood flow from the central retinal artery is blocked. The results of experimental studies on aged hypertensive and atherosclerotic monkeys suggest that the survival of the ischemic retina extends to 240 min (8). Other findings indicate that complete retinal infarction develops after only 15 min (9). Nevertheless, CRAO recanalization treatment similar to that for occluded arteries in other body parts can be applied. A recently performed meta-analysis suggests a benefit in visual outcomes if intravenous thrombolysis is administered within 4.5 h (6). Another crucial aspect of the proper and timely diagnosis of CRAO is revealing its sources to allow appropriate interventions to prevent unwanted health-associated events, including stroke or restoration of the unaffected eye in the case of CRAO due to inflammation. For example, in the case of stroke prevention after experiencing a retinal stroke, it is recommended to apply fast (within 24–72 h) diagnostics (10).

## 3 Retinal stroke as carotid artery ischemic stroke

The central retinal artery is a branch of the ophthalmic artery (derived from the internal carotid artery) that supplies the inner layer of the retina with blood. Embryological and histological peculiarities of the retina with brain tissue and common risk factors for acute cerebrovascular and retinal ischemia formed the basis for the inclusion of the retina in transient ischemic attack and renewed ischemic stroke definitions in 1975 and 2013, respectively (11). International epidemiological stroke studies currently include CRAO and retinal stroke under the umbrella of ischemic stroke (12).

The main causes of CRAO are embolism or hemodynamic insufficiency due to diseases of the cardiovascular system, such as atherosclerosis, arrhythmia, or abnormalities of the valvular or cardiac wall. A less common cause (<5% of cases) of retinal stroke is arteritic CRAO (7). CRAO is associated with newly diagnosed ischemic stroke in >5% of all patients 15 days before or after the accident (13), whereas acute silent brain infarction on magnetic resonance tomography imaging (MRI) was found in 27 to 76.4% of all CRAO cases (14, 15). Furthermore, a recent retrospective investigation from Korea revealed a > 7 times increase in standardized mortality ratio after CRAO, mainly due to cardiovascular and cerebrovascular causes, compared to the general population (3). In this context, the American Heart Association and the American Academy of Ophthalmology recommend the triage of these patients to specialized stroke centers for treatment, investigation, and follow-up (14, 16). A recently published study highlighted the importance of multidisciplinary assessment in the case of retinal stroke because it revealed increased death, stroke, and myocardial infarction after CRAO compared to matched control both in the short and long term, respectively (17).

Currently, although multiple treatment methods have been described for acute non-arteritic CRAO (thrombolysis, eyeball massage, and electrical stimulation, among others), a Cochrane systematic review reported that no single treatment is effective compared to observation alone; however, the quality of evidence is low (18). Despite these findings, thrombolysis is the most promising treatment for non-arteritic CRAO (19). We hope that the uncertainties regarding thrombolysis will be answered by the results of ongoing trials.^1^ The REVISION (Early Reperfusion Therapy With Intravenous Alteplase for Recovery of VISION in Acute Central Retinal Artery Occlusion) and THEIA (THrombolysis (Alteplase) in Patients With acutE Central retInal Artery Occlusion). Currently, up to 14.1% of non-arteritic CRAO cases are treated (off-label) with intravenous thrombolysis (20); however, timely patient arrival at an emergency room with a stroke service is paramount for this treatment to be an effective and appropriate preventive measure.

## 4 Managing CRAO in the emergency room

Patients with CRAO showing monocular vision loss as an ophthalmological emergency may be presented to the emergency room by a paramedic team or are self-referred; however, more commonly, these patients are referred by an ophthalmologist, optometrist, or general practitioner. A study in Switzerland reported that 47.7% (167 subjects from 350) of the surveyed population in the St. Galen hospital outpatient clinic with unassisted questionnaires would consult an ophthalmologist or general practitioner upon experiencing acute monocular loss of vision (21). Another part of this study from the stroke register showed that only 17% of those who experienced retinal stroke arrived at the neurovascular center by ambulance, compared to 65.3% of ischemic stroke cases. Furthermore, 41% of all CRAO cases were referred from other hospitals, likely due to limited neurological and ophthalmological resources in the primary hospital (21). It is important to emphasize that 57.45% (74 of 129) of responding hospitals with stroke units from Germany lacked emergency ophthalmological assessment on-site (22). As awareness of retinal stroke emergencies improves in Western countries, more patients with CRAO arrive promptly at the emergency department with stroke services (20). Unfortunately, many patients still do not have the opportunity to receive potential recanalization treatment because of late arrival or delayed diagnosis (23). According to the guidelines, patients who arrive directly at the emergency department with suspected acute CRAO must be examined by an ophthalmologist to confirm or exclude other causes of monocular blindness (14). However, the ophthalmological investigation is time-consuming, and often, neither the ophthalmologist nor the necessary equipment are available for comprehensive on-site investigation. Moreover, fundoscopic examination by an ophthalmologist does not always reveal typical CRAO fundoscopic findings, such as the “cherry red spot sign” and artery attenuation, as described in the 19th century by Von Graefe A., including (24), especially in the very acute period (7). Moreover, fundoscopic examination often cannot directly reveal emboli in the retinal arterial tree (25–27). The most specific CRAO fundoscopic “cherry red spot sign” is present in 55.71–90% of cases (28, 29). A normal fundus appearance is observed in 14% of cases (30). “Cherry red spot sign” describes retinal edema in the macular region. Edema formation takes time—early CRAO (especially within the first 4.5 h) will likely show weak or no retinal edema, as established in a recent study with applied optical coherence tomography (OCT) investigation (31). Furthermore, the gold standard for CRAO ophthalmological instrumental investigation, fluorescein angiography, may show normal findings in 26.67% of CRAO cases (30). Another ophthalmological tool for CRAO diagnosis is OCT-angiography (OCT-A) (32). The main advantage of OCT-A is its ability to provide a detailed assessment of retinal microvasculature without the need for dye injection, with the possibility of detecting changes earlier than with conventional OCT (28). Furthermore, OCT-A has the potential for the detection of retinal penumbra through the quantification of collaterals (7). The disadvantage of OCT-A is the dependence on long fixation (it is more difficult in CRAO cases with impaired vision) for clear images (28).

However, ophthalmological examination has some merits: it can help to reveal other causes of acute monocular and painless loss of vision, including retinal detachment, massive vitreous hemorrhage, and vasculitic changes in the case of arteritic CRAO (33). Furthermore, fundoscopic inspection aids the prognosis of CRAO. For example, cases with a congenital variant of the cilioretinal artery have a better prognosis, with improvement in vision in 67% of cases (7).

Another method for the evaluation of retinal supply vessels in suspected cases of acute CRAO is ultrasound, which is usually performed in contemporary emergency room settings (34–36).

## 5 Ultrasound applications in acute painless monocular blindness

More than 20 years ago, ultrasound in the emergency room was established as a reliable ophthalmological work-up for discriminating CRAO, retinal detachment, and massive vitreous hemorrhage (37).

Recently, a working group of different European neurosonological organizations has released Neuro-POCUS, a conjoint document for point-of-care ultrasound (POCUS) applications in acute neurological conditions (38). In this document, the streamlined bedside examination includes a transorbital ultrasound to monitor intracranial pressure and evaluate the blood vessels supplying the eye. One critically important practical application of Neuro-POCUS is the detection of eye emergencies, such as CRAO (39). Currently, Neuro-POCUS is performed by an ultrasonographer who is competent in performing and interpreting data gathered during nervous system investigations by ultrasound, including the hemodynamic diagnosis of retinal ischemia (38). This practice is usually performed by neurologists (neurosonologists) in certain countries. Ultrasonography has advantages over computer tomography (CT) or MRI because it is applicable on-site, inexpensive, provides information on brain and eye hemodynamics instantaneously, does not require contrast, sedation, or radiation, and can effortlessly and rapidly (<5 min) diagnose CRAO (38, 40).

Ultrasound examination of eye vasculature is performed using color-coded sonography with a high-frequency linear transducer after minimization of the mechanical index to ≤0.23 and thermal index ≤1 to avoid potential damage to the eye constituents according to the principle of “as low as reasonably achievable” (41). The central retinal artery enters the optic nerve approximately 13 mm behind the eyeball and travels parallel to the central retinal vein; therefore, these vessels can be easily found and evaluated after guiding the ultrasound beam near the end of the optic nerve-peripapillary region (42). The blood vessels passing through the optic nerve, specifically, the central retinal artery and vein, can be easily distinguished using Doppler waves, with more pulsating arteries and more continuous venous flow in opposite directions. Flow in the central retinal artery should be measured by applying sample volume in the center posterior to the lamina cribrosa, using an adapted setting for “low flow,” without correcting the measurement angle (43). During routine examinations, it is important to evaluate other retro-orbital arteries, such as the posterior ciliary artery, which supplies the choroid and optic nerve disk and runs parallel to the optic nerve, as well as the deeper ophthalmic artery. For detailed information on these examinations, refer to ref. (42).

In isolated CRAO, persisting flow is typically observed in the central retinal vein but not in the artery. A common ultrasound finding in such cases is the detection of hyperechogenic material near the end of the optic nerve, known as a “positive spot sign.” This sign indicates a lodged calcified or crystalized cholesterol embolus in the central retinal artery behind the lamina cribrosa (5, 44), a finding that has demonstrated excellent intra-observer agreement (45). Rare cases show a traveling “spot sign” from the proximal to distal parts of the central retinal artery that clinically progresses from a transient loss of vision to blindness (46). Case 1 shows a case of CRAO with a “positive spot sign” located more proximally in the central retinal artery than is typical. The findings consistent with the “positive spot sign” perfectly exclude arteritic CRAO (45, 47), thus helping to avoid unnecessary steroid administration in cases with elevated levels of inflammatory markers during the acute CRAO period (48). This sign is detected in 31–83% of CRAO cases (5) and is associated with a significantly reduced probability of recanalization and worse visual outcomes (49). However, it does not theoretically exclude the potential for recanalization (6). Thus, transorbital ultrasound could add potential value for differentiating candidates for thrombolytic therapy based on visualized embolic materials (50). “Spot sign negative” CRAO encompasses a broader range of reasons, including central retinal artery thrombosis *in situ*, embolism, hemodynamic insufficiency, compression, and vessel wall inflammation or spasm.

**CASE 1**

**Figure 1:**
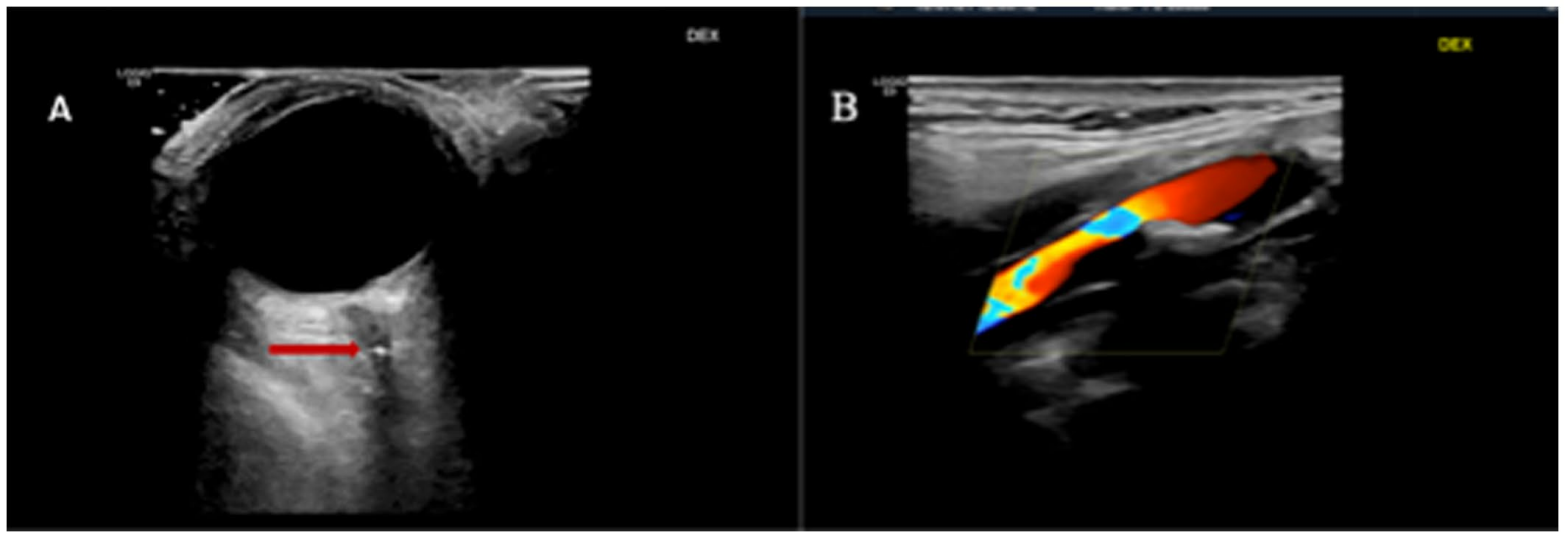
**(A)** Transorbital ultrasound of the right eye with a “positive spot sign” proximal to the optic nerve head in the central retinal artery (arrow) and **(B)**, right symptomatic carotid stenosis.

A 60-year-old man presented with acute and painless loss of vision 260 min from the onset of the symptom to the emergency room. Neuro-POCUS revealed a “positive spot sign,” but much more proximally in the central retinal artery than usual (Figure 1A). Additionally, carotid ultrasound revealed moderate ipsilateral carotid stenosis (Figure 1B). Secondary prevention, in addition to medical therapy, consisted of carotid endarterectomy.

Case 2 shows an example of the practical application of transorbital ultrasound in a case of acute CRAO with thrombolytic treatment. Critical aortic valve stenosis is a potential cause of retinal stroke.

**CASE 2**

**Figure 2:**
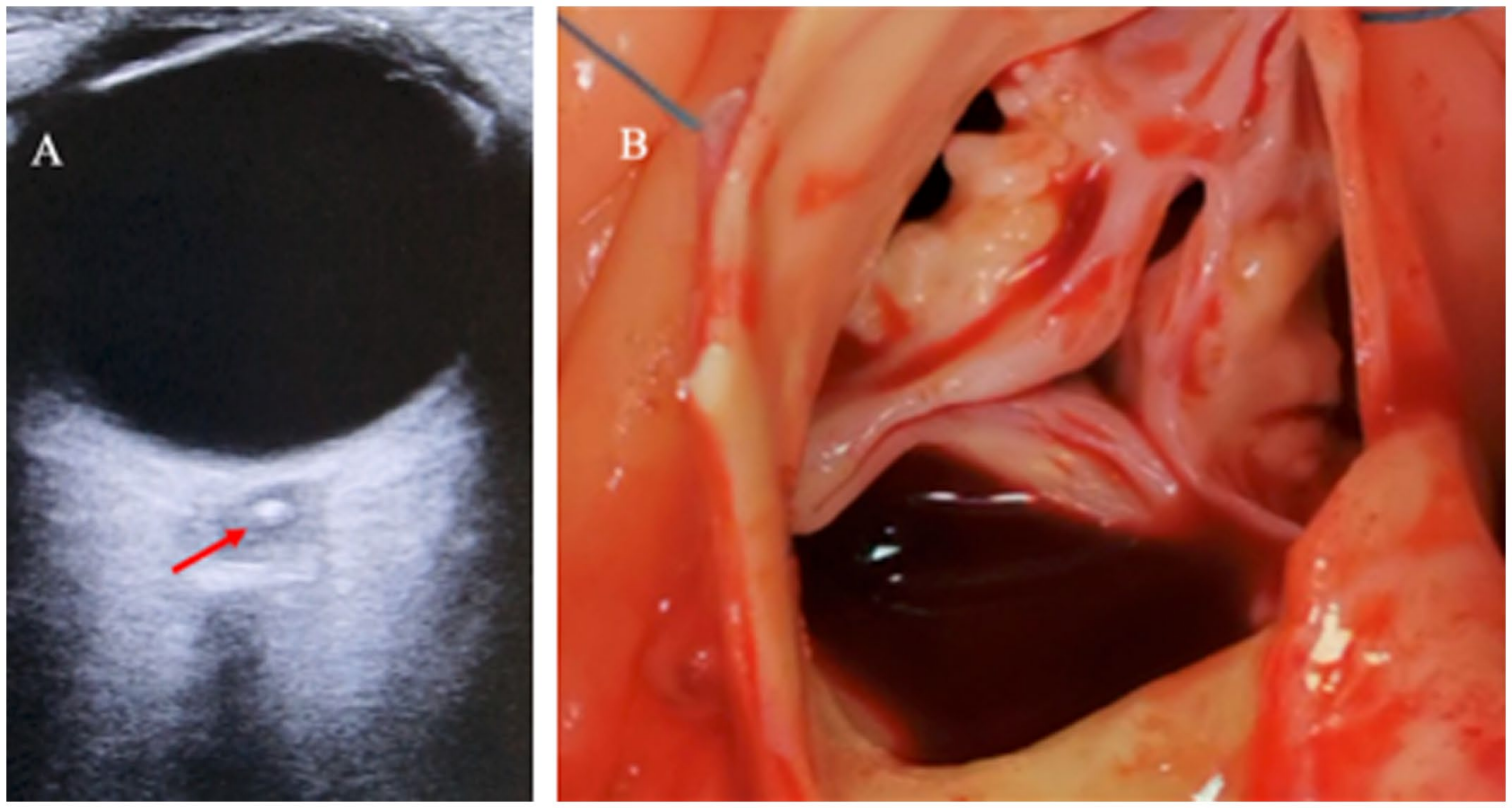
**(A)** Transorbital ultrasound of the right eye with a “positive spot sign” in the optic nerve head (arrow) and **(B)** calcified stenotic aortic valve during cardiac surgery.

A 70-year-old woman who had an anamnesis of hypertension and several episodes of consciousness impairment during the last year presented with sudden total blindness of the right eye after bending forward to wash her head. First, the patient was referred by the paramedic team to an ophthalmologist an hour after the onset of the blindness. Fundoscopic examination revealed typical signs of the ischemic retina—"positive cherry red spot” and opaque whitening. She was referred to an emergency room with stroke service. On-duty neurosonologists performed Neuro-POCUS on cervical and retro-bulbar arteries. During the examination, a “positive spot sign” with no flow in the central retinal artery was found to be consistent with the CRAO diagnosis of the right eye (Figure 2A), with bilateral carotid artery stenosis near 50%. A head CT revealed no significant changes. CT angiography of the brain-supplying vessels confirmed the findings of the cervical ultrasound. Neurological examination did not show any other neurological sign, except for monocular blindness (The National Institutes of Health Stroke Scale score was zero). After excluding contraindications to intravenous thrombolysis, with patient consent, treatment with alteplase was initiated after 2 h from the onset of the symptom. During the follow-up, no vision improvement appeared during the next 24 h. Additional etiological work-up revealed critical stenosis of the aortic valve with possible cardioembolism of the calcific material to the central retinal artery. Two weeks later, cardiac surgery was applied (Figures 2B). Total blindness of the right eye and a “positive spot sign” on ultrasound persisted 4 weeks after symptom onset.

Another condition, non-arteritic anterior ischemic optic neuropathy (AION) with impaired optic nerve head blood flow (supplied by posterior ciliary arteries), could present with sudden and severe loss of vision in one-tenth of cases (51). Albeit transorbital ultrasound in non-arteritic AION could reveal reduced blood flow in the central retinal artery and temporal posterior ciliary arteries (52), it is an unreliable method to discriminate against this condition caused by impaired optic nerve microcirculation (53).

The added value of ultrasound is its utility in the diagnosis of a rare cause of arteritic CRAO, namely, giant cell arteritis, which requires distinct anti-inflammatory treatment for the restoration of the unaffected eye. Arteritic CRAO should be suspected in older patients presenting with jaw claudication, new-onset headaches, and temporal tenderness. In addition, elevated levels of inflammatory blood markers are also often observed. Giant cell arteritis in the orbit has a preference for the ophthalmic artery and its branches (54). Most commonly, giant cell arteritis manifests as arteritic AION and less commonly as arteritic CRAO (55). In arteritic AION, transorbital ultrasound reveals no flow in the posterior ciliary arteries (56). Ultrasound is a standard tool for the diagnosis of giant cell arteritis based on temporal artery evaluation (57). In this case, ultrasound helps to visualize inflammatory infiltration of the temporal artery, which presents as a hypoechogenic “halo” sign visible in transverse and longitudinal planes. Another important sign of giant cell arteritis revealed by the temporal artery ultrasound evaluation is a positive “compression” sign—an uncompressible artery with an ultrasound probe (57). Transorbital ultrasound is also able to diagnose other causes of painless and sudden visual loss, such as retinal detachment (rope-like intraocular membrane) and massive vitreous hemorrhage (the presence of materials with different echogenicity within the eyeball) (58). However, other causes of monocular vision loss, such as uveitis or acute maculopathy, and other ophthalmological conditions are not described here (33).

The limitations of ultrasound in assessing eye-supplying vessels are associated with unreliable blood flow volumetric measurements and the inability to identify vessel wall morphology (59). In the case of the “positive spot sign,” this observation should not be confused with the hyperechogenic calcified mass or drusen visible between the optic nerve and retina (60). With ultrasound “B” (Brightness) mode drusen are visible in the optic nerve head, while calcified embolus or “positive spot sign” is usually behind it, but sometimes even within it (39, 45, 61). In addition to this, in the case of drusen, the flow in the central retinal artery persists and is visible with the ultrasound Doppler regime, albeit with an increased resistance index, at least in bilateral involvement (62).

It is also important to note that ultrasound assessment is operator-dependent and requires sufficient experience and workload (>15 examinations for intermediate skills) to gain and maintain competency (38).

## 6 Neurosonologist’s perspective on retinal stroke

CRAO is a complex eye-threatening condition with various causes and treatment strategies that should be considered from a multidisciplinary perspective for treatment success (63). Ophthalmologists, neurologists (63), cardiologists (cardiac embolization of the central retinal artery), and rheumatologists (in case of vasculitic disorder) should be included. In our opinion, during the acute retinal stroke period in cases with suspected acute CRAO, if the symptom onset is <4.5 h, an experienced neurologist/neurosonologist (combining knowledge of stroke diagnostics, treatment, and prevention with practice in Neuro-POCUS application) being in the emergency room could: (1) rapidly diagnose CRAO occlusion on-site with POCUS; (2) reveal the potential CRAO causes by investigating the cervical carotid arteries, intracranial hemodynamics, and temporal arteries in cases of suspected vasculitis with the same POCUS for the timely application of preventive measures; and (3) determine patient eligibility for recanalizing treatment according to local protocols. While waiting for the ophthalmologist in case of suspected acute CRAO, which could potentially be suitable for treatment with thrombolysis, the neurologist/neurosonologist should perform brain CT and exclude possible contraindications for this treatment (16). At this point, only after an ophthalmological assessment should a final decision be made on thrombolytic therapy. In this case, the neurosonologist’s use of transorbital ultrasound examination may be useful in shortening the time to the final decision on retinal stroke-specific treatment with lytic agents. Whether neurosonological testing using ultrasound could replace ophthalmological testing in retinal stroke can only be answered after further research in the future.

Since review articles with information on practical ultrasound applications in diagnosing retinal ischemia are lacking (7, 14, 16), we agree with the other authors that the message regarding the possible practical utility of Neuro-POCUS in diagnosing CRAO requires wider dissemination (64). Furthermore, one in three ophthalmologists reported that they would not transfer their patients with CRAO to a stroke center (21), thus leaving the patients at risk of future stroke or other cardiovascular conditions. This attitude has motivated us to spread current knowledge and stimulate new research to evaluate the possibility of replacing ophthalmological examinations with Neuro-POCUS for the diagnosis and treatment of acute CRAO. This could be particularly beneficial in remote areas or lower-middle-income countries where emergent ophthalmological work-ups are unavailable.

## 7 Conclusion

We suggest that transorbital ultrasound has added value in detecting acute CRAO if performed by a well-organized interdisciplinary team in a neurovascular center with stroke expertise, and we attest that it is a useful adjunctive to an ophthalmological investigation. Additionally, it is helpful in the identification of potential causes of CRAO. Subsequently, it can guide clinicians in implementing optimal secondary prevention strategies. We accept that further research is needed before recommending lytic agents to patients with non-arteritic CRAO, solely based on the findings of transorbital ultrasound.

## Data availability statement

The datasets presented in this article are not readily available because of ethical and privacy restrictions. Requests to access the datasets should be directed to the corresponding author.

## Ethics statement

Ethical review and approval was not required for the study on human participants in accordance with the local legislation and institutional requirements. Written informed consent from the patients/participants or patients/participants' legal guardian/next of kin was not required to participate in this study in accordance with the national legislation and the institutional requirements. Written informed consent was obtained from the individual(s) for the publication of any potentially identifiable images or data included in this article.

## Author contributions

ST: Writing – original draft. DJ: Writing – review & editing. VM: Writing – review & editing. SR: Writing – review & editing. JV: Writing – review & editing, Conceptualization.

## Footnotes

^1^https://clinicaltrials.gov
